# Röntgen Ray and Its Usefulness1Read before the Buffalo Medical Union, February 23, 1898.

**Published:** 1898-06

**Authors:** Frederick Preiss

**Affiliations:** Buffalo, N. Y., Lecturer in electro-therapeutics.


### RÖNTGEN RAY AND ITS USEFULNESS.[1]

By FREDERICK PREISS, M. D., Buffalo, N. Y.,
Lecturer in electro-therapeutics.

TO THOROUGHLY familiarise yourself with the discovery of the Röntgen ray, I shall give you a summary of experiments which led up to this important event. Faraday invented the terms anode and cathode, which indicate the conductor terminals of a current of electricity. He also studied the effects of electrical discharges within tubes containing rarefied gases. Geissler improved these tubes and increased the degree of rarefication; he also experimented with many kinds of gases, noticing the beautiful effect of a number of them. It was also noted that these gases acted differently at the anodal and cathodal terminals within the tubes and that fluorescence was produced, which was the result of the cathode extremity. Following these experiments came the magnificent researches of Prof. Crookes, who, by his high vacuum tubes, demonstrated that electrified particles were projected in straight lines within the tubes from the cathode end, producing a fluorescence of the glass, which was caused by the bombardment of these electrified particles.

Next came Hertz, who showed that the cathode rays possessed penetrable power within the tube, and his student, Lenard, discovered that the cathode rays possessed the same qualities outside the tube to about the distance of three inches from the tube, and that the ray would pass through certain substances easier than through more dense objects; he also showed that these shadows caused by the ray not passing though opaque substances might be impressed on a sensitive plate and developed in the usual art of photography. But to Prof. Röntgen is given the credit of producing similar effects at enormously long distance from the tube, he also being the first to bring the ray into practical use by having shadow-photographs taken of the bones of the human organism. Prof. Röntgen claimed that the rays from which these results were obtained were not those of his predecessors and brought forth arguments to substantiate his claim, but arguments may be brought forward also in favor of the cathode ray being identical with the Röntgen ray, differing from it only in degree as regards severity or penetrable power. In my opinion the ray is cathodal and is developed in any of the Crookes or Geissler tubes by the passage of electricity through them, and the strength or penetrable power of the ray depends wholly upon two favorable conditions—namely, (1) a cer-

1. Read before the Buffalo Medical Union, February 23, 1898.

tain amount of electricity of high electromotive force; (2) the proper vacuum of the tube used. After many experiments I have come to the conclusion that the Hertz, Lenard and Röntgen ray are all one and the same, differing only in the degree of penetrable power, as above explained.

There has not been any discovery in any line of science which has caused as much worldwide interest as has Prof. William Conrad Röntgen's discovery of the properties of the penetrating light commonly called the *X*-ray. That name, in my opinion, is inappropriate for the following reasons—namely, in the first place the letter "*X*" is made use of in difficult problems to represent an unknown quantity and that is why it has been made use of in this instance. Although the ray is somewhat obscure, still we know that it is a light and is produced by the passage of electricity of very high voltage through a glass tube which has been previously exhausted to 1-1,000,000 part of air; consequently, when we know the origin, development and properties, I am not in favor of having it represented by the letter "*X*", but am more in favor of calling it after the discoverer of its usefulness and who was instrumental in introducing it to be used in a practical way. This personage is Prof. Röntgen.

My object in this paper will be to give a concise description of a Röntgen ray apparatus and describe its usefulness and, inasmuch as this subject is somewhat new and much experimentation is going on at the present time, I shall avoid, as far as possible, all unnecessary technical terms and theoretical discussions. Before advancing further on this subject I shall explain a few terms which I shall make use of.

(*a*) A "volt" is a practical unit of electro-motor force; (*b*) an "amperé" is a practical unit of rate of speed; (*c*) the "cathode" is a name given to the negative pole terminal; (*d*) the "anode" is a name given to the positive pole terminal; (*e*) a "Leyden jar" is composed of glass and has a tin-foil coating inside and outside of the jar to about one-half its height; a cork stopper is used through which a brass rod runs, having a brass chain attached to its inner end, which touches the tin-foil, and a brass ball or ring attached to its outer end; the inner tin-foil is called the internal armature and the outer tin-foil is called the external armature, (*f*) high vacuum or high density is a name applied to the Crookes tube when it requires great electromotor force to drive the electricity through the tube in order to give the best penetrable light; (*g*) low vacuum or low density is a name applied to the Crookes tube when less electromotor force is required to drive the electricity through the tube and the consequent penetrating power of the ray is much less than that of the high vacuum tube; (*h*) the fluoroscope is an instrument of great importance to the operator of the Röntgen ray. This instrument was devised by Prof. Edison and is composed of a pasteboard screen, covered with either fused tungestate of calcium or barium platino-cyanide, set in a pyramidal box, having this screen as the bottom. This screen serves to the operator the same as the lens does to the telescope manipulator. Prof. Edison has experimented with many different salts, but up to the present writing the barium platino-cyanide is by far the superior to any yet discovered for use in conjunction with the Röntgen ray. By the use of this instrument the operator is enabled to tell with what degree of perfection his tube is being excited and so aid him greatly in shadow-photography, or an examination may be made by the use of this instrument without the trouble or expense of having a shadow-photograph taken. It is a common practice of the operator of the Röntgen ray apparatus to test the penetrable power of his tube by looking through his hand and noticing the distinctness of the bony outline. I am much opposed to such a practice, as injurious effects may sooner or later develop if he uses the coil apparatus to a considerable extent. I should advise a metallic or other object to be placed in a book or box and to be looked at each time he operates, and soon his eye will become accustomed as to the clearness of the object when the tube is working to perfection. This method, as I advise, is equally instructive and not at all injurious to the manipulator.

In the development of the Röntgen ray there are three main apparatuses used to excite the Crookes tube—namely, (1) the static machine; (2) the induction coil; (3) the Tesla transformer.

In order to do Röntgen ray work from a static machine the size of the machine must first be taken into consideration. As a rule, an eight plate or more does the best work, although the ray can be obtained from as small a machine as a four plate, but not with any satisfactory results. There are three methods employed to excite the tube by a static machine: (*a*) the convective, (*b*) the interrupter spark gap, (*c*) the Leyden jar oscillating current. The only difference in all these methods is in the connection. A description of each is here given. (1) In the convective method, simply connect the tubes to the prime conductors and be sure that you have connected the anode of the static to the anode of the Crookes tube, and in commencing operation by this method have the spark-gap two inches between the sliding terminals and gradually pull them apart beyond sparking space while the machine is in motion. It is customary to place large Leyden jars beneath the pole pieces of the static machine in the hope that better results may be obtained, the jars acting as condensers and having a tendency to reinforce the current; but I have not noticed any material difference in such arrangements. (2) The Leyden jar oscillating current is attached by connecting the tube terminals with Leyden jars having not more than twelve square inches to the external or internal armatures; otherwise, if larger jars are used, the condensation is so great and the consequent current reinforced to such an extent that injury may be done to the tubes. In commencing operation by this method have the sliding poles close together and gradually pull them apart beyond sparking capacity as the machine is being worked. In having the Leyden jars in a circuit, remember that by induction the current is changed—namely, the prime conductor giving positive electricity and entering the internal armature as such, is negative when it leaves the external armature; therefore, for example, an anodal prime conductor of the static machine is attached to the cathodal end of the Crookes tube, providing the Leyden jar is in the circuit between the static machine and the tube. (3) The interrupter spark-gap is connected in the following manner: having first noted the anodal and cathodal terminals and having placed the interrupters on the handles of the sliding rods, which have been pulled wide apart, connect the anodal interrupter to the anodal terminal of the tube and the cathodal interrupter to the cathodal terminal of the tube. In commencing operation with a machine connected in this manner have the interruptions about one-eighth of an inch in space and gradually increase this space to about one inch at the positive and to one-half inch at the negative pole. Of course, this space of spark-gap will depend greatly upon the size and density of the tube. The Leyden jars may be in their proper position with the external rod extending high enough to be in contact with the post of the sliding terminal. In this position the jars are supposed to act as condensers and so increase the electromotor force of the current. This method of connecting I claim to be superior to the other two, for the following reasons: (1) There is not as much waste of current and consequently a greater amount passes through the tube; (2) the interrupters give greater bombardment to the ray within the tube and thereby greater penetration is produced.

The induction coil is the most convenient, especially if the apparatus be taken to the bedside or clinic room. The first object which confronts the purchaser, however, is how large a coil should be bought? For all ordinary purposes a six to ten inch is sufficient; it also must be decided whether a direct or alternating current is to be used for the primary circuit or battery. I should recommend the direct current in the form of a movable battery. A necessary accessory to the coil is a vibrator or rotary interrupter; both work satisfactory, but a rotary interrupter run by a small motor is to be preferred, as more even interruptions are thereby obtained, which is conducive to better Röntgen ray production. A rheostat should be used to control the current supplied to the primary of the induction coil, as, if too strong a current be passed to the coil, it is very liable to be burnt out and consequently ruined. If the coil be immersed in oil it is not so easily short-circuited and will give the purchaser more service.

*Tesla Transformers.*—Mr. Tesla has devised a coil which develops statical electricity and may be attached to a direct or alternating current and consequently may be connected to any of our electric light currents. Mr. Tesla claims that with an ordinary incandescent lamp his coil may be used in place of the ordinary static machine in the treatment of various diseases. Unfortunately the coil is not manufactured at present, but Mr. Tesla informs me, however, it will be in the course of a few months. Such an apparatus would be very useful, as it could be carried very easily to the bedside or the clinic room, where electricity or a small battery is at our command, without the slightest inconvenience, as the whole apparatus would not weigh over twenty pounds.

Many names have been given to the pictures taken in conjunction with the Röntgen ray. The following is a partial list: Cathode-photography, shadowgraphy, radiography, cathography, photography, electrography, fluorography, skiagraphy and röntography. There are two methods whereby this picture is taken: (1) by putting the object which is to be shadow-photographed between the sensitive plate and the Crookes tube; (2) by having a fluoroscopic screen and putting the object between this screen and the Crookes tube, and then with a camera take the picture of the image or shadow which appears on the screen.

You will observe that in either case we do not get a photograph of the object itself, but a photograph of the shadow of the object is produced; therefore, I have designated the word shadow-photography, which is self-explaining and is an appropriate word to use in conjunction with this part of the Röntgen ray work. In giving a description of shadow-photography the process is identical, whether a small or large apparatus be used, or whether the shadow-photograph is to be that of a bone or that of a foreign body. Now, supposing the experimenter is ready to proceed. He takes the plate-holder containing the sensitive plate, the film side of which is turned upward, and fixes the object between the tube and the sensitive plate; everything being in readiness the apparatus is made to work. The length of time required for the exposure depends upon the following conditions: (1) the penetrating power of the rays; (2) the amount of tissue or substance which the ray will be required to penetrate.

There are now on the market plates wrapped in black paper and a plate-holder is not required. These plates will keep from four to six months without any deterioration as regards their sensitiveness. The experimenter must always remember that the Röntgen ray will destroy the sensitive plates; consequently these plates must be kept in an iron box or in an adjoining room having a partition made of other than carbonous material. Furthermore, the object to be shadow-photographed must be kept perfectly quiet, otherwise a fogging of the picture will result. You are all familiar with what a photographer will say when you have a photograph taken—namely, keep quiet and do not move a muscle; so in a shadow-photography, the object must be kept perfectly quiet. If the object be fastened to the sensitive plate it does not matter so much if both move slightly, but one must not move differently than the other. If you wish, for instance, to take a shadow-photograph of the hand, fasten the hand firmly to the plate by three or four bands of adhesive plaster. To the beginner, questions arise, (1) how far should the plate be kept from the tube? That depends upon the apparatus you have and the power of the ray, but, as a rule, very good results are obtained at about two to six inches from the tube. At this distance the picture will be the best as regards accuracy and fine definition. (2) How long must the sensitive plate be exposed? That depends upon two conditions: (*a*) The density of the tube, whether high or low; if the density be low, much time is required and at its best the definition is poor and inaccurate; but if the density be high, a much shorter time is requisite to obtain a perfect impression, a few seconds or even an instant of exposure to a perfect flash is worth more than minutes of the working of the ordinary tubes of low vacuum. (*b*) The object the ray has to penetrate, as, for example, under like conditions it will take double the time for the elbow than for the hand. There are a few suggestions I shall endeavor to make here in regard to the manipulation of the tube. To the experimenter it is of the utmost importance to have the tube working perfectly before the sensitive plate is exposed, and herein the fluoroscope is made use of, after you have turned on the apparatus; have a certain object to look through and see whether the tube is working at its best. If you accustom yourself to look at the same object each time you will soon familiarise yourself as to how plain the object should appear when the tube is at its best, whereas if you use a different object each time you will have lost that advantage.

A word about the Crookes tube. This tube is exhausted to 1-1,000,000 part of air, having at each end a platinum wire fused in the glass and ending externally in a loop to make attachments to the exciting apparatus. Internally these wires end differently; the one is attached to a dish, usually made of aluminum, which function is to concentrate the rays and is called the cathodal extremity of the tube. It is always attached to the negative pole of the exciting apparatus, the other being attached to a reflector and is usually made of the same material; its function is to reflect the rays and is called the anodal extremity, and is always attached to the positive pole of the exciting apparatus. If these attachments be reversed, little or no penetrating ray will be detected outside the tube. If the vacuum of the tube be too high, heat the cathodal extremity slightly over a spirit lamp, taking care not to heat it too much at one point, as you are liable to break the tube by so doing. If the vacuum be too low, use the tube a while and the vacuum will gradually get better and the ray more penetrable. After the tube has been in use considerable and is not working perfect, reverse the connections a few minutes and the tube will again probably work to perfection; an impaired tube is also benefited by rest; but after a time the tube cannot be benefited by this means of repairing and will have to be sent to the manufacturers for reëxhaustion. Many tubes are on the market and it depends upon what kind of exciting apparatus it is to be used, as regards which kinds of tubes are best suited for that particular outfit. In connecting the tube with the exciting apparatus I should recommend the connecting link to be made of fusible lead wire, as a more perfect connection can be made and consequently less injury done to the tube through manipulation while making the necessary attachments. Tubes are manufactured which contain a salt in an extension at one end of the tube. This salt can be heated from outside the extension and lower the vacuum if it be too high. This tube is commonly called a focusing tube.

Great has been the interest taken by all the educated human race in the achievements of the Röntgen ray, and the class that has been most interested is that of the medical profession, who are ever eager to grasp at new remedies and appliances that may assist them in their efforts to relieve diseased, suffering humanity. Among the foremost revelations of the Röntgen ray are those applied to normal anatomy and the day is not far distant when a first-class Röntgen ray apparatus will, out of necessity, be among the paraphernalia of the dissecting laboratory of every foremost medical college and hospital of the universe. It might be argued that the student can study as well from an artificially arranged skeleton, but such is not the case, as no human hand can arrange the osseous structures to the same perfection as nature. By the Röntgen ray shadow-photograph or by the use of the fluoroscope the precise relations of the bones to each other may be determined when the body is in the erect position or in any of the various attitudes. Development may be studied with great advantage, as developing bone may be easily distinguished from that which has already developed; likewise, the comparative anatomist is furnished an opportunity to study the osseous structures of the lower animals.

In the dissecting room the anatomical relations of the blood-vessels may be accurately determined by injecting into the vessels of the cadaver a metallic or nonpenetrable substance, which will show by opaqueness the precise course and distribution of the arterial circulation; the feasibility of this method may also be applied to the various cavities and organs of the dead body. In the living subjects the dimensions of the stomach may be determined by having the patient swallow ferruginous pills, or better still, a metallic ball attached to a string or flexible handle, and a shadow-photograph taken and the fluoroscope used while the patient is in certain positions. Irregularities and congenital deformities of the osseous structures may easily be determined; also the heart, liver and the kidneys may be outlined. To the surgeon the Röntgen ray is as requisite as the mirror is to the laryngologist or the ophthalmoscope is to the oculist. In considering the diseases of the bony structures of the human organism we find the ray indispensable in various pathological conditions, most of which I shall endeavor to bring before you. How often are we consulted when, owing to the extreme tenderness and extensive swelling, thereby causing inability to properly manipulate the disabled member, we are unable to accurately decide whether the case at hand is one of fracture, dislocation, a severe sprain with much laceration of the soft tissue, or perhaps all three; but now with the use of the Röntgen ray we may very easily determine the exact nature of the existing disability and treat our patient with confidence and not with the fear of a possible malpractice suit. If in case of fracture you manipulate the broken bones and apparently get them in proper position, but are still in doubt as to whether the ends of the bones are in perfect apposition, all that is requisite is to place your subject before the ray and with the use of the fluoroscope you will be able to satisfy yourself whether or not they are now in their natural position. If the splints be carbonous, you may at intervals look through them and determine whether the bones are kept immovable and that healing is going on properly.

We find the ray also very useful in determining tubercular and cancerous disease of the bone, caries and necrosis, exostosis, floating cartilage of osseous formation and hypertrophy of bone; in fact, any disease whatever which shows increase or loss of bony substance. Many times we are consulted for troublesome pains referable to the bony structures. So localised is the pain that the patient is strongly under the impression that the bone is affected. By the use of the ray you will be able to convince your patient that such is not the case, but that the symptoms complained of are those caused by a probable localised neuritis and treat the patient accordingly. And so in a great many instances where the patient imagines that something is wrong with the osseous structure, you have only to use the ray, which will aid in the diagnosis and also gain the confidence of the patient and so aid materially in your endeavor to relieve the existing troublesome condition.

It is also very important to diagnosticate whether anchylosis, caused by a fracture or disease, is due to fibrous or bony union, inasmuch as the former may be remedied with good success and the latter not. The ray, in passing through an anchylosed joint, due to fibrous union, will show a light space between the ends of the bones; if due to bony union this space will appear opaque. Consequently the surgeon is in a position to enlighten his patient on the probable result if operative procedure be performed in either case.

In dentistry the ray is occasionally made use of in detecting whether or not a fang of a tooth remains in the socket, even if it be covered with soft tissue. The surrounding bone is more penetrable than are the teeth, thereby distinguishing the alveoli from the teeth or fangs. The lost end of a broken drill may be located if the dentist unfortunately breaks his instrument while operating upon his patient; also the central cavity of a tooth may be outlined so that diseased conditions within the tooth may be detected. The growth and development of the teeth may be studied before and after they begin to protrude above the gums, thereby greatly aiding in the diagnosis of certain obscure cases of convulsions occurring during infantile teething.

One of the first applications of the discovery of Prof. Röntgen was the detection of foreign objects in the human body. Many occasions we meet with cases where the patients have been injured by fire-arms, whether intentionally or otherwise, and no one knows better than the surgeon how difficult it is sometimes to locate the bullet, but with the use of the Röntgen ray the exact position may be manifested, and once the object being located there are three methods by which to proceed for its removal:

1. The surgeon may take a glance through the fluoroscope to see where the foreign body is located and mark the spot, then giving the fluoroscope to his assistant. He takes his knife or forceps, as the case may require, and operates; if necessary he may be directed by his assistant, who has the management of the fluoroscope and is watching the operation through it.

2. A shadow-photograph may be taken whereby the exact location of the foreign body is determined and the operator, having the picture before him, can now very easily extract the object.

3. By the use of the fluorometer, which is by far the best method of exactly locating any foreign object. Not only does this apply to missiles sent by fire-arms, but to all substances which are nonpenetrable to the ray, such as needles, glass, pieces of steel, stone and the like. Then, again, we find important diagnosis made by the rays in abdominal concretions, such as stone in the kidney, bladder and liver. Also calcareous deposits may be detected in various parts of the body, such as in gouty and rheumatic affections.

Suppose you are consulted by a patient who has had articular rheumatism for many years, you will be unable in the majority of cases to say whether the swelling around the joint is all external to the periosteum, but by the use of the ray you may very easily determine enlargement of the osseous structure and consequently be able to give a more satisfactory diagnosis and prognosis of the case at hand. Fibroid growths of large dimensions, whether simple or malignant, may be outlined. Pregnancy may be diagnosticated as soon as the fetal osseous structures become slightly nonpenetrable to the ray. Prior to four months' gestation it is almost useless to attempt diagnosis by the above means.

To the practising physician the ray is not of such great importance as it is to the surgeon, but it may be made use of by him to determine consolidation of the lung, enlargement of the liver, enlargement of the kidney, enlargement of the uterus, displacement and enlargement of the heart, and is useful in the diagnosis of disease by exclusion. In almost all other diseases the practising physician will find the Röntgen ray of little service to him.

In the foregoing pages I have endeavored, to the best of my ability, to lay before you, in a concise way, the pathological and anatomical conditions wherein the use of the Röntgen ray is of great importance. Of course, I could cite many instances where I have found the ray of incalculable service. An example is here given:

January 8th a brother physician brought a patient of his to me for a Röntgen ray examination, with the following history: Pain more or less for six years since falling off a platform; at the time of the accident he alighted on both feet. At that time he was confined to the house for four days, after which he was out and about by the aid of a cane. Upon making a comparative shadow-photograph of both knees the bone of the injured leg, which was the lower end of the femur, was found to be half an inch wider in lateral diameter than the other; consequently, at the time of the accident the lower end of the femur must have been slightly cracked and was never properly put together. He was informed at this examination that nothing could be done and was much disappointed, but was pleased to be enlightened as to the exact nature of the existing difficulty. Previous to this he had consulted many physicians without any relief or satisfaction. Now, under the circumstances it would have been impossible to have diagnosticated this condition by any other advisable method except the one I have described.

Another very important application of the Röntgen ray will be in connection with testimony in lawsuits. Up to this date only two cases are on record where the presiding judge allowed the above used as such and in these cases it was used only as corroborative testimony. In all other cases the court did not allow such testimony as might be given by shadow-photography, but it will only be a matter of time when such evidence will be permissible. The main reason why shadow-photograph testimony is not at the present date always permitted is because most of the cases in the court at the present time are the result of injuries sustained before the ray was discovered and consequently the defendant did not have the advantage of the use of this recent discovery.

I may say that the field of experimentation of Röntgen ray work is large. There is ample room to make use of the ray in other directions than it has been used up to the present time. It is an interesting and notable fact that a diamond can easily be distinguished from a paste or glass, as the latter will appear opaque while the former will not. It may also be used to detect flaws in metals or how much metal there is in certain ores. Who knows but that in a short time, by chemical or mechanical devices used in conjunction with the Röntgen ray, we will be able to differentiate between the cyst, hematoma or a collection of pus and the like.

A word here about the beneficial and curative effect of the Röntgen ray. Allow me to say that, in my opinion, it has not any such property whatever. Much experimentation has been carried on, but all without the slightest encouraging result, but, on the other hand, injurious effects may be procured by a constant or repeated exposure of a coil apparatus.

Cases have been cited of the falling out of the hair, erythematous sloughing, inflammation of the eyelids and skin generally, and falling off of the nails, but this injury, in my opinion, is not produced by the ray itself, but is produced by the subject being placed in too close proximity to the electric current, which current is of high voltage and consequently the patient is subjected to some extent to the electro-galvanic burning, which manifests itself in and around the tube and its conductors. I have yet to witness a single case where the slightest injurious effects have been produced by the proper manipulation of the Röntgen ray apparatus; but if operated by an imprudent or unskilful hand, occasionally considerable injury may be manifested. Pray, what is not injurious if carried to excess?

The ray itself appears to have very little, if any, action upon animal or vegetable organisms. Any action that does manifests itself is due to electric diffusion, which is the result of leakage from the tube and its conductors while the apparatus is being operated.

160 FRANKLIN STREET.

